# KMP01D Demonstrates Beneficial Anti-inflammatory Effects on Immune Cells: An *ex vivo* Preclinical Study of Patients With Colorectal Cancer

**DOI:** 10.3389/fimmu.2020.00684

**Published:** 2020-04-30

**Authors:** Martin Gasser, Reinhard Lissner, Karol Nawalaniec, Li-Li Hsiao, Ana Maria Waaga-Gasser

**Affiliations:** ^1^Department of Surgery I, University of Wuerzburg, Wuerzburg, Germany; ^2^Department of Surgery I, Molecular Oncology and Immunology, University of Wuerzburg, Wuerzburg, Germany; ^3^Renal Division, Brigham and Women's Hospital and Harvard Medical School, Boston, MA, United States

**Keywords:** immune regulator, KMP01D, vitamin D3, colorectal cancer, inflammation

## Abstract

**Background:** Colorectal cancer (CRC) is frequently associated with dysbiosis of the gut microbiome which, together with a compromised gut barrier, can result in perioperative endotoxin leakage into the circulation. Constant local and systemic inflammatory activity is suggested to facilitate metastases formation. Previous studies have pointed to the capacity of a colostrum preparation to neutralize endotoxins within the gastrointestinal tract which could ameliorate associated inflammatory responses and tumor recurrence in affected patients. This study aimed to examine the effects of the colostrum preparation, KMP01D, on the inflammatory activity of patient-derived immune cells.

**Methods:** The effects of KMP01D on pro-/anti-inflammatory cytokine responses and apoptosis were examined *ex vivo* using immune cells from CRC patients (stages I–IV, *n* = 48). The expression of CD14, CD68, Toll-like receptor (TLR)4, and insulin-like growth factor (IGF)-1 was also analyzed.

**Results:** KMP01D increased interleukin (IL)-10 and IL-13 anti-inflammatory cytokine expression in patient-derived peripheral blood mononuclear cells (PBMCs). Interestingly, KMP01D also decreased the secretion of IL-1β, IL-6, interferon (IFN)-γ, tumor necrosis factor (TNF)-α, IL-12 inflammatory cytokines, and IGF-1 in these cells. Moreover, CD14 and TLR4 expression involved in endotoxin signaling was downregulated in PBMCs and tumor-derived cells. Apoptosis of immune cells and tumor-derived cells was likewise enhanced with KMP01D. Addition of vitamin D3 as a cofactor demonstrated enhanced anti-inflammatory effects.

**Conclusions:** KMP01D demonstrated beneficial *ex vivo* effects on inflammatory cytokine responses in PBMCs and enhanced apoptosis of immune cells from CRC patients. In line with previous clinical trials, we present new evidence endorsing KMP01D as a treatment strategy to regulate stage-dependent local and systemic inflammation in CRC patients.

## Introduction

According to the World Health Organization, overall survival in colorectal cancer (CRC) patients is ~40–50%, making this disease the third most common cancer-related cause of mortality in men and the second in women in Western societies. First-line treatment for CRC has been surgical removal of the tumor followed by chemo- or radiochemotherapy. (Neo) adjuvant combination regimens of 5-fluorouracil/leucovorin and oxaliplatin (FOLFOX) or irinotecan (FOLFIRI) have been used for patients with lymph node-positive stage III CRCs, and the mean 5-year survival has improved to over 60% in patients from Western societies according to the Union for International Cancer Control (UICC). The additional implementation of monoclonal antibodies in clinical protocols to specifically inhibit angiogenesis in patients with UICC stage IV disease with liver metastases has likewise improved the mean 5-year survival. Despite this, the overall survival for advanced stage IV cancers with distant metastases remains limited at only 20–30%. Even following tumor removal, a significant group of stage III (resection of the primary tumor) and IV (primary tumor and metastasis) CRC patients does not effectively benefit from (neo) adjuvant treatment protocols. As such, these patients are susceptible to developing metastatic tumor growth and progression despite the use of modern chemotherapeutics and biologics.

Previous clinical and preclinical studies have evidenced that an inflammatory micro- and macro-environment in primary and secondary metastatic tumors in patients with CRC supports the migration process of tumor cells and their seeding in distant organs such as the liver and the lungs (1, 2). Furthermore, growing evidence suggests that perturbations of the immune system promote tumor progression and have negative impact on prognostic indicators such as time until tumor recurrence and overall patient survival (3–7). With no anti-inflammatory strategies currently available for these patients, great focus has been placed on the underlying inflammation-mediated mechanisms involved in a disturbed innate and adaptive immune response.

Intestinal microbiota and the immune system within the gut interact with one another to maintain a homeostatic balance in healthy individuals. An imbalance of gut microbiota, particularly toward gram-negative bacteria, results in the production of endotoxins [i.e., lipopolysaccharides (LPS)]. The homeostasis between the integrity of the intestinal epithelial barrier and low dose but repeated exposure to endotoxins becomes disturbed in patients with underlying malignancy, Crohn's disease, chronic kidney disease, advanced age, and impaired immune editing (3, 8–12). Patients with CRC often exhibit a defective epithelial gut barrier and dysregulation of their gut microbiota which promote endotoxin influx into the circulation and leads to intermittent low-grade or constant local and systemic inflammation.

Endotoxins bind Toll-like receptor (TLR)4 and CD14 on monocytes/macrophages, triggering their activation and an inflammatory response (13, 14). Such a disturbance of the immune system results in dysregulation of inflammatory cytokine levels and alteration of apoptosis of monocytic cells. Interestingly, *Escherichia coli*, a gram-negative bacterial strain often dysregulated in the epithelial gut layer of CRC patients, has been shown to specifically prevent phagocytosis-induced death of macrophages via classical anti-apoptotic nuclear factor (NF)-κB signaling and activation of these cells (15). Thus, an imbalanced gut microbiome, particularly within the expanding tumor of the bowel wall, promotes local and systemic inflammation and can likewise impair apoptosis of monocytes and macrophages (16). Additionally, increasing evidence has demonstrated a low lymphocyte-to-monocyte ratio (LMR) to be associated with shortened overall survival in various malignancies including CRC. This has recently been summarized in a meta-analysis of more than 20,000 patients with solid cancers (4). Endotoxin neutralization at the epithelial surface may therefore contribute to restoration of immune cell apoptosis, increasing LMR, and decreasing inflammatory cytokine levels. Such mitigation of disturbed inflammatory activity in UICC stage I–III CRC patients may help to prevent residual tumor cells from metastasizing after primary surgery.

Based on principles of naturally transferred immunity in breast-fed newborns, the intake of first-milk, or colostrum, is a natural process by which the newborn acquires the immune characteristics of the mother (“acquired” or “transferred immunity”). Administration of a colostral preparation containing polyvalent immunoglobulins (PVIGs) with O-chain specificity against potentially harmful gram-negative bacteria has recently gained attention in the context of inflammation. PVIGs have shown anti-inflammatory potential in neutralizing endotoxins in the gut and mitigating related inflammatory processes (17–23). Two placebo-controlled clinical studies have previously shown that oral administration of colostral immunoglobulin preparations (Lactobin) significantly reduced endotoxin leakage into the circulation and resulted in diminished expression of inflammation markers in patients (21, 22). Clinical studies in infants with enterohemorrhagic *E. coli* (EHEC) infection and in adults with severe viral infection have likewise provided evidence that the orally administered colostrum preparation (Lactobin) effectively neutralizes endotoxins in the gastrointestinal tract (24). PVIGs in combination with other colostral components (e.g., lipoprotein-binding proteins, vitamins, lactoferrin) are thus thought to have the potential to correct perturbations of the immune response (e.g., TLR4/CD14 signaling pathway) known to occur in cancer, particularly in CRC (2, 21–25).

**Figure 1:**
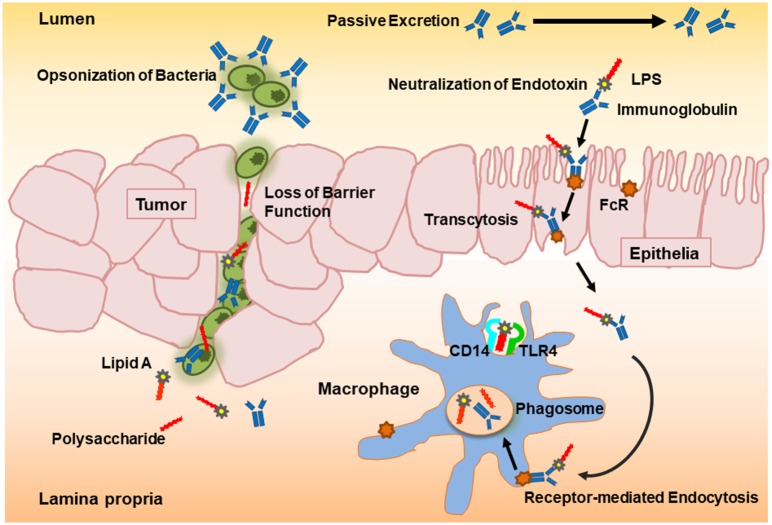
Schematic overview of the proposed neutralization potential of polyvalent immunoglobulins (PVIGs) found in KMP01D. Orally ingested colostral PVIGs of bovine origin pass through the gut lumen where they bind and thereby neutralize endotoxin [lipopolysaccharide (LPS)] or opsonize bacteria (such as *Escherichia coli*). As unmodified and therefore foreign proteins, processing of orally administered PVIGs proceeds either by passive excretion or internalization of receptor-bound PVIGs by monocytes/macrophages following transcytosis or leakage through the epithelial layer.

Orally administered colostral PVIGs pass through the gut lumen where they bind and thereby neutralize endotoxins. Although a partial proteolytic degradation of the protein molecules takes place in the upper gastrointestinal tract when administered in a non-encapsulated form, up to 50% of the ingested PVIGs can be recovered in feces (17, 26–28). PVIGs likewise bind the Fcγ receptors on human mucosal cells and submucosal macrophages in the gut and can thereby become internalized in phagosomes and proteolyzed to peptides in phagolysosomes through the process of receptor-mediated endocytosis (16, 17, 29, 30). Hence, as unmodified and therefore foreign proteins, processing of orally administered PVIGs proceeds by either passive excretion or receptor-mediated internalization (Figure 1).

Evidence from both epidemiological and experimental studies shows that higher intake or endogenous levels of vitamin D3 supports a reduced risk of CRC (31, 32). Vitamin D binds the vitamin D receptor expressed in various cell types including colorectal cells and enables the transactivation of target genes that promote cellular differentiation, induce apoptosis, and inhibit angiogenesis and proliferation. The vitamin D receptor and vitamin D-mediated signaling have been implicated in colorectal carcinogenesis. As such, we sought to analyze a potential additive effect of vitamin D3 when used in combination with KMP01D.

In this study, we investigated, for the first time, the *ex vivo* effects of KMP01D (i.e., LactobinN) on the inflammatory activity of immune cells derived from UICC stage I–IV CRC patients. KMP01D was analyzed alone and in combination with vitamin D3 for its effects on cytokine expression and immune cell apoptosis.

## Materials and Methods

### Patient Cohort and Human Tissue Samples

Prospectively enrolled patients undergoing elective surgery for primary CRC at the University Hospital of Wuerzburg, Germany, were included in this study. All patients of UICC stages I–IV (*n* = 48; mean age of 66.1 ± 5.6 years) underwent curative R0 (indicating microscopically margin-negative) resections, with UICC stage IV patients undergoing additional resection of their distant metastases. Patients with secondary carcinoma were excluded. All patients were followed up regularly for 60 months or until death in accordance with the guidelines of the German tumor centers (completeness index of 0.96). The study was approved by the Regional Ethics Committee of the University Wuerzburg and performed in accordance with the ethical principles stated by the Declaration of Helsinki. Patient samples were cataloged in the Tumor and Sera Bank together with completed follow-up data from the database of the Comprehensive Cancer Center at the University of Wuerzburg, a government-funded institution in Germany conforming to Organization for Economic Cooperation and Development (OECD) Guidelines. Tumors were evaluated for location, stage, and differentiation grade. Data concerning age, gender, and post-operative course with follow-up were additionally collected in our database. Tumor tissue and normal colon tissue samples and blood from these patients were collected with informed consent. For the analysis of tumor-derived cells, tumor tissues were dissociated and placed in a collagenase solution, incubated at 37°C, and washed with RPMI 1640 medium (Gibco, Eggenstein-Leopoldshafen, Germany), and necrotic cells and debris were removed by density gradient centrifugation. Isolated cells were then counted, frozen, and stored in liquid nitrogen until analysis. Additionally, 30 ml of peripheral blood was collected from each patient before surgery. Peripheral blood of healthy volunteers (*n* = 12) was collected as controls. After collection, EDTA tubes were left to stand at room temperature for 60 min. Plasma was then isolated and stored at −80°C. Peripheral blood mononuclear cells (PBMCs) were separated by Ficoll-Hypaque (Amersham Biosciences AB, Freiburg, Germany) density gradient centrifugation. PBMCs were washed in RPMI 1640 medium containing 4% normal human plasma (Gibco) and stored in liquid nitrogen before analysis.

### KMP01D and Vitamin D3

The colostrum preparation, KMP01D (i.e., LactobinN), was provided by Dr. Wolz Zell GmbH, Geisenheim, Germany. KMP01D is comprised of over 80% protein by weight, <2% of fat, <11% of lactose, <5% of water, and <0.2 μg/100 g of vitamin D (average values). In addition to PVIGs (particularly of the IgG1 class), KMP01D contains cytokines, growth factors, and enzymes. For IgGs (subclass IgG1), quantitatively measured values are 21–24% by weight. A recommended daily oral dose of the preparation is 10–20 g according to a limited number of existing clinical studies using KMP01D. The ready-made preparation was used in this study. To determine the optimal dose of KMP01D to be used in our *ex vivo* experiments, a full dose/response analysis was first performed and compared with the neutralization capacity dose of KMP01D (data not shown). PBMCs or tumor-derived cells from disintegrated primary tumor tissues of CRC patients as well as PBMCs from healthy volunteers were incubated with KMP01D, vitamin D3, or the combination of both (10 mg KMP01D and 0.025 μg vitamin D3/5 × 10^5^ cells/ml) for 24 h in RPMI 1640 medium supplemented with 10% human serum, 1% penicillin/streptomycin, 1% 4-(2-hydroxyethyl)-1-piperazineethanesulfonic acid (HEPES), 1% non-acid, 1% Na-pyruvate, and 1% L-glutamine (Biochrom, Berlin, Germany). These culture conditions were maintained for all experiments.

### Flow Cytometric Analysis

Isolated PBMCs were incubated with KMP01D, vitamin D3, or the combination of both for 24 h and analyzed for monocyte frequency. Cells were stained using anti-CD14-phycoerythrin (PE), anti-CD45-fluorescein isothiocyanate (FITC), and 7-aminoactinomycin D (7-AAD) viability dye. Staining was performed according to the manufacturer's instructions. Samples were analyzed by a flow cytometer (Beckman Coulter, Krefeld, Germany) with a software package (Coulter, Epics XL-MCL, System II). Each data sample was analyzed by first gating according to forward scatter and side scatter to isolate monocytes, lymphocytes, and granulocytes and to exclude debris. This heterogeneous population was then selected for viable 7-AAD-negative cells and further analyzed according to the expression of CD14 and CD45 markers where CD14^+^CD45^+^ events were considered monocytic. The percentage of monocytes in each sample was determined by setting a gate on the CD14^+^CD45^+^ population according to untreated control samples and transferring this gate to all patient samples.

### Gene Expression Analysis

Gene expression in PBMCs and tumor-derived cells was analyzed by extraction of total RNA followed by reverse transcription and quantitative real-time PCR (RT-qPCR). The following biomarkers were analyzed: insulin-like growth factor (IGF)-1, CD14, CD68, and TLR4. RNA was extracted from PBMCs and homogenized tumor tissue using the RNA Extraction Kit (Qiagen, Hilden, Germany), washed in diethyl pyrocarbonate (DEPC) 75% ethanol solution, and stored at −80°C. The amount of RNA was determined by measuring absorbance at 260 nm. The 260:280 ratio was determined within a range of 1.8–2.0. The primers were designed using the Primer Express Software for primer design to produce short amplicons of 100–200 base pairs of the defined cDNA. The quantitative real-time PCR was carried out using 11.5 μl of LightCycler DNA Master SYBR Green I mix (Applied Biosystems, Darmstadt, Germany) with 2 μl of cDNA (50 ng/μl), 0.3 μM forward primer, and 0.3 μM reverse primer to give a total reaction volume of 23 μl. The PCR cycle consisted of a 15-min initial denaturation at 95°C, followed by 40 cycles of 15-s denaturations at 95°C, a 30-s annealing step at 55–61°C, and a 3-s extension step at 72°C. Gene-specific products were continuously measured using the ABI Prism 7700 sequence detector (Applied Biosystems, Foster City, CA), and the relative expression was calculated. Analysis of all samples was carried out in duplicates. The amplification curves measured for the individual gene products were depicted graphically and evaluated in relation to the curve of the control genes. To quantitatively determine the target template, the average CT value (threshold cycle) was measured, that is, the number of cycles within which the fluorescence of the reporter reaches a fixed threshold above the baseline values. We then calculated the difference (ΔCT) between the average CT values of the samples in the defined wells and those of the housekeeping genes, glyceraldehyde-3-phosphate dehydrogenase (GAPDH) and β-actin. The difference (ΔΔCT) between the average ΔCT values of the samples for each target gene and the ΔCT value of the corresponding control (PBMCs: healthy volunteers, tumor-derived cells: normal colon tissue) for the target gene was calculated next. This relative quantification value (also called fold difference) was expressed as 2^ΔΔCT^.

### Cytokine Analysis Using Luminex

The human cytokine bead assay kit (Biosource, Nivelles, Belgium) was used for the detection of cytokines [interleukin (IL)-1β, IL-6, interferon (IFN)-γ, tumor necrosis factor (TNF)-α, IL-12, IL-10, and IL-13] in supernatants of cultured PBMCs (5 × 10^5^ cells) in response to KMP01D, vitamin D3, or the combination of both. The assay was performed according to the manufacturer's instructions. Analysis was carried out in duplicates using the Luminex 100 instrument (Biosource). Results are expressed in pg/ml.

### Quantitative Determination of Human Insulin-Like Growth Factor-1

To detect IGF-1 present in culture supernatants of PBMCs, we used an enzyme-linked immunosorbent assay (ELISA; Biosource International, San Diego, CA) in accordance with the instructions from the manufacturer. The results were read by an ELISA reader (Dynatech Laboratories, Sullyfield, USA) at 450 nm. Results are expressed in ng/ml.

### Immunofluorescent Staining

Cytospin preparations of cultured PBMCs and tumor-derived cells were completed by washing cells twice with phosphate buffered saline (PBS; Life Technologies, Carlsbad, CA), adjusting the cell suspensions to a concentration of 2 × 10^5^ cells/ml, and centrifuging 50 μl of cell suspension at 550 rpm for 1 min using a cytocentrifuge (Thermo Fisher Scientific, Waltham, MA). Cytospins were incubated with the primary or isotype control antibody followed by incubation with the corresponding FITC-conjugated secondary antibody. Cytospins were subsequently incubated with the second primary or isotope control antibody followed by incubation with the corresponding cyanine 3 (Cy3)-conjugated secondary antibody. Cytospins were counterstained with 4′,6-diamidino-2-phenylindoldihydrochloride (DAPI; Sigma-Aldrich, Steinheim, Germany), covered with polyvinyl-alcohol mounting medium [1,4-diazabicyclo[2.2.2]octane (DABCO); Sigma-Aldrich], and analyzed using a Zeiss microscope (Zeiss, Oberkochen, Germany). The quantification of each immunofluorescent staining was performed by two independent investigators blinded for the underlying disease. The chosen magnified fields were representative for the whole cytospin.

### *In situ* Detection of Apoptosis Using Terminal Deoxynucleotidyl Transferase dUTP Nick end Labeling Assay

A non-isotopic DNA end-labeling *in situ* technique employing digoxigenin-dNTP and terminal transferase was used to identify cells with fragmented DNA (ApopTag® Plus Peroxidase *in situ* Apoptosis Detection Kit, Merck Milipore, Darmstadt, Germany) as described (23). Terminal deoxynucleotidyl transferase dUTP nick end labeling (TUNEL)/CD68 double staining was performed to identify and enumerate apoptotic CD68^+^ PBMCs and tumor-derived cells. Apoptosis of cells was identified by TUNEL-positive staining of the whole nuclear area. The degree of apoptosis in each treatment group was observed at a magnification of 400 × in at least 10 high-power fields.

### Statistical Analysis

Statistical analysis was performed using GraphPad Prism 5.0 (GraphPad Software Inc., San Diego, CA). A two-way ANOVA with Bonferroni *post hoc* test or a one-way ANOVA with Bonferroni *post hoc* test was used according to sample distribution. For non-parametric data, the Kruskal–Wallis and Mann–Whitney U tests were used. All experiments were conducted at least three times, and the results are expressed as the mean ± standard deviation. *p* < 0.05 was considered statistically significant. For comparative analysis of cytospins of PBMCs and tumor-derived cells, positively stained cell counts were determined by two independent investigators using three individual high-power fields (400×).

## Results

### The Combination of KMP01D and Vitamin D3 Decreased Monocyte Frequency in Peripheral Blood Mononuclear Cells of Patients With Colorectal Cancer

**Figure 2:**
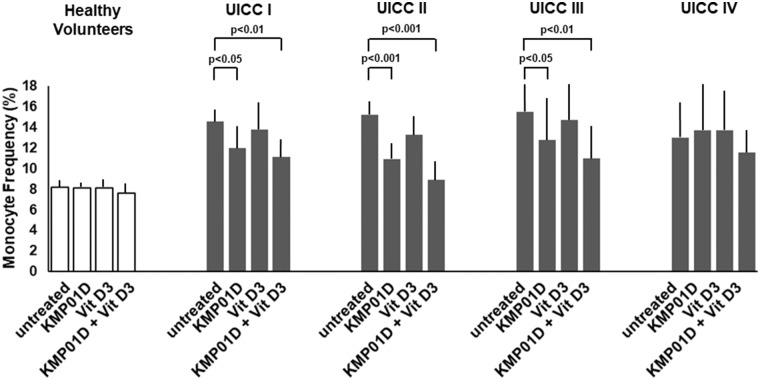
Comparative analysis of monocyte frequency (CD14^+^CD45^+^ cells) determined by flow cytometric analysis of peripheral blood mononuclear cells (PBMCs) from healthy volunteers (*n* = 12) and Union for International Cancer Control (UICC) stage I–IV colorectal cancer (CRC) patients (*n* = 12 for each stage). Decreased expression of CD14^+^CD45^+^ cells was observed in the PBMCs of UICC stage I–III CRC patients incubated with KMP01D and the combination of KMP01D and vitamin D3 (UICC I: *p* < 0.05 and *p* < 0.01, UICC II: *p* < 0.001 and *p* < 0.001, UICC III: *p* < 0.05 and *p* < 0.01, respectively).

**Figure 3:**
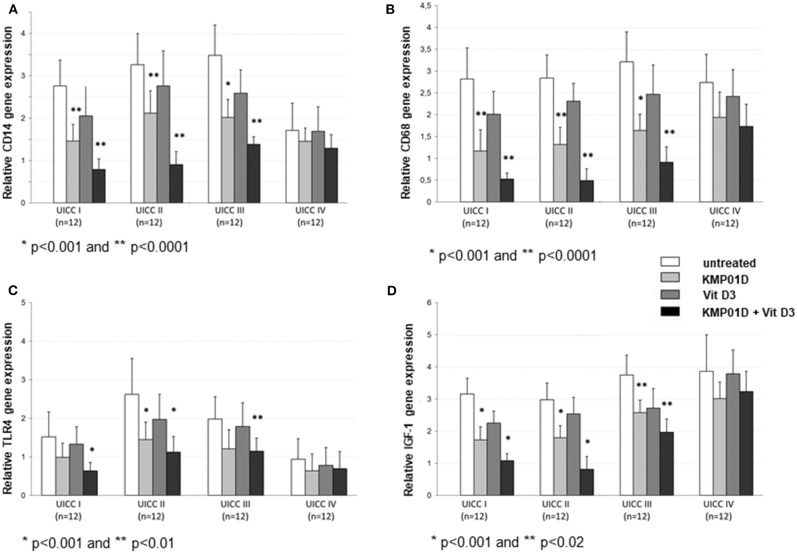
RT-qPCR analysis showing decreased **(A)** CD14, **(B)** CD68, **(C)** Toll-like receptor (TLR)4, and **(D)** insulin-like growth factor (IGF)-1 gene expression in peripheral blood mononuclear cells (PBMCs) from Union for International Cancer Control (UICC) stage I–III colorectal cancer (CRC) patients (*n* = 12 for each stage) following incubation with KMP01D and the combination of KMP01D and vitamin D3 compared with untreated cells (CD14: UICC I–II: ^**^p < 0.0001 and ^*^p < 0.0001, UICC III: ^*^p < 0.001 and ^*^p < 0.0001; CD68: UICC I–II: ^**^p < 0.0001 and ^*^p < 0.0001, UICC III: ^*^p < 0.001 and ^*^p < 0.0001; TLR4: UICC I: *p* > 0.05 and ^*^p < 0.001, UICC II: ^*^p < 0.001 and ^**^p < 0.001, UICC III: *p* > 0.05 and ^**^p < 0.01; IGF-1: UICC I–II: ^*^p < 0.001 and ^*^p < 0.001, UICC III: ^**^p = 0.02 and ^**^p = 0.02, respectively). An enhanced effect was observed after incubation with the combination of KMP01D and vitamin D3.

Monocytes play a critical role in the cross-talk between inflammation-mediated lymphocytes and cancer cells in tumor progression. Incubation of PBMCs with the combination of KMP01D and vitamin D3 demonstrated enhanced effects on decreasing monocyte frequency in isolated PBMCs of patients with early and advanced stage tumors (UICC stages I–III) (Figure 2, Supplementary Figure 1). Furthermore, the combination of KMP01D and vitamin D3 mitigated CD14 gene expression in patient-derived PBMCs which was shown to be highly significant in UICC stages I–III (*p* < 0.0001; Figure 3A). Interestingly, CD68 gene expression was likewise decreased in these cells, both with KMP01D alone and in combination with vitamin D3 (KMP01D vs. untreated cells: UICC I–II: *p* < 0.0001, UICC III: *p* < 0.001; combination of KMP01D and vitamin D3 vs. untreated cells: UICC I–III: *p* < 0.0001; Figure 3B). Incubation of patient-derived PBMCs with the combination of KMP01D and vitamin D3 yielded a more effective decrease in CD14 and CD68 expression than KMP01D alone (Figures 3A,B). In addition, gene expression of TLR4 co-receptor, important for conveying LPS pro-inflammatory signaling, was also suppressed by the combination of KMP01D and vitamin D3 (UICC I–II: *p* < 0.001, UICC III: *p* < 0.01; Figure 3C).

### The Combination of KMP01D and Vitamin D3 Decreased the Expression of Inflammation-Related Cytokines, Chemokines, and Growth Factors in Cultured Peripheral Blood Mononuclear Cells From Colorectal Cancer Patients and Promoted Immune Cell Apoptosis

Pro-inflammatory cytokine (IL-1β, IL-6, IFN-γ, TNF-α, and IL-12) expression was upregulated in untreated PBMCs of CRC patients when compared to healthy controls (UICC I–II: IL-1β, IL-6, IFN-γ: *p* < 0.001, TNF-α, IL-12: *p* < 0.01, UICC III–IV: IL-1β, IL-6, IFN-γ, TNF-α, IL-12: *p* < 0.001). In contrast, anti-inflammatory cytokine (IL-10 and IL-13) expression was shown to be downregulated relative to healthy controls (UICC I–IV: *p* < 0.01). IGF-1 was stage-dependently upregulated in CRC patients (UICC I: *p* < 0.01, UICC II–IV: *p* < 0.001). Incubation of PBMCs from UICC stage I–III CRC patients with KMP01D and the combination of KMP01D and vitamin D3 decreased IL-1β, IL-6, IFN-γ, TNF-α, and IL-12 cytokine expression which was observed in parallel with decreased CD68, CD14, and TLR4 expression in these cells (UICC I–II: IL-1β, IL-6, IFN-γ, TNF-α, IL-12: *p* < 0.01, UICC III: IFN-γ, TNF-α, IL-12: *p* < 0.001 and IL-1β, IL-6: *p* < 0.05, UICC IV: IFN-γ, IL-12: *p* < 0.01; Table 1). Furthermore, our results demonstrated decreased expression of IGF-1 after incubation of PBMCs with KMP01D, vitamin D3, and the combination of both (KMP01D vs. untreated cells: UICC I–II: *p* < 0.001; UICC III: *p* = 0.02; combination of KMP01D and vitamin D3 vs. untreated cells: UICC I–II: *p* < 0.001, UICC III: *p* = 0.02; Figure 3D). Comparison of PBMCs incubated with KMP01D alone and in combination with vitamin D3 suggested additive effects in patients of UICC stages I–III (*p* < 0.05; Table 1, Figure 3D).

**Table 1:** Cytokine expression from PBMCs after incubation of KMP01D and/or vitamin D3.

	**PBMCs**	**IL-1β pg/ml**	**IL-6 pg/ml**	**IFN-γ pg/ml**	**TNF-α pg/ml**	**IL-12 pg/ml**	**IL-10 pg/ml**	**IL-13 pg/ml**	**IGF-1 ng/ml**
**Healthy volunteer (*****n*** **=** **12)**	Untreated	4.7 ± 0.4	3.1 ± 0.6	12.6 ± 2.3	62.1 ± 10.3	47.8 ± 6.5	27.5 ± 4.2	43.3 ± 9.3	93.4 ± 6.3
	KMP01D	4.5 ± 0.6	3.0 ± 0.2	12.3 ± 3.1	62.2 ± 14.2	49.1 ± 7.1	27.8 ± 4.7	44.1 ± 11.2	91.4 ± 5.4
	Vit D3	4.8 ± 0.1	3.3 ± 0.3	12.8 ± 4.7	62.6 ± 10.7	47.7 ± 8.3	27.9 ± 5.8	43.6 ± 10.6	90.3 ± 4.7
	KMP01D + Vit D3	4.5 ± 0.4	3.0 ± 0.1	12.1 ± 5.2	62.2 ± 10.3	41.1 ± 6.9	28.1 ± 4.8	44.9 ± 9.5	89.1 ± 4.1
**UICC I (*****n*** **=** **12)**	Untreated	60.1 ± 7.8	99.2 ± 10.8	71.8 ± 6.3	120.1 ± 6.5	97.6 ± 8.3	11.9 ± 6.4	23.0 ± 7.3	195.2 ± 20.5
	KMP01D	21.9 ± 7.4	38.8 ± 9.4	42.3 ± 7.5	72.6 ± 7.4	61.9 ± 8.1	24.7 ± 6.2	33.9 ± 8.2	122.3 ± 22.6
	Vit D3	42.5 ± 6.8	98.7 ± 9.5	58.5 ± 9.3	83.6 ± 6.3	74.9 ± 9.6	18.3 ± 7.4	30.1 ± 8.9	148.6 ± 24.1
	KMP01D + Vit D3	11.3 ± 6.3	41.9 ± 7.4	30.4 ± 7.6	64.8 ± 7.3	51.0 ± 9.5	26.9 ± 8.3	42.9 ± 8.4	95.3 ± 19.3
**UICC II (*****n*** **=** **12)**	Untreated	58.1 ± 8.6	113.2 ± 5.8	103.9 ± 11.6	223.4 ± 8.4	113.5 ± 12.6	10.4 ± 3.6	17.1 ± 9.7	261.7 ± 27.3
	KMP01D	20.8 ± 7.5	76.3 ± 6.4	57.3 ± 10.7	121.7 ± 10.2	69.4 ± 10.4	20.9 ± 4.2	36.6 ± 9.5	186.7 ± 32.3
	Vit D3	34.8 ± 7.9	102.8 ± 8.9	77.3 ± 11.1	169.1 ± 11.1	80.1 ± 12.8	18.1 ± 3.1	27.9 ± 10.3	213.4 ± 33.6
	KMP01D + Vit D3	15.6 ± 6.1	81.6 ± 5.9	46.2 ± 8.6	86.3 ± 9.3	59.6 ± 9.7	23.9 ± 3.8	39.3 ± 7.4	101.8 ± 47.2
**UICC III (*****n*** **=** **12)**	Untreated	175.0 ± 10.1	95.4 ± 11.7	258.9 ± 8.3	362.8 ± 10.7	305.7 ± 21.8	7.8 ± 4.3	10.1 ± 4.9	334.8 ± 30.4
	KMP01D	147.5 ± 9.5	73.5 ± 9.4	165.0 ± 10.9	293.1 ± 11.4	175.3 ± 23.6	20.1 ± 3.5	31.4 ± 4.5	249.9 ± 44.2
	Vit D3	169.4 ± 14.7	91.7 ± 7.6	215.9 ± 9.4	331.5 ± 11.9	214.7 ± 19.4	17.9 ± 7.8	26.1 ± 5.8	299.1 ± 61.1
	KMP01D + Vit D3	118.1 ± 7.8	76.7 ± 4.3	150.1 ± 10.8	264.7 ± 10.5	92.9 ± 19.7	22.9 ± 6.3	35.8 ± 6.7	183.5 ± 33.2
**UICC IV (*****n*** **=** **12)**	Untreated	372.8 ± 15.6	213.2 ± 11.1	335.7 ± 9.5	423.4 ± 9.3	532.2 ± 14.2	5.0 ± 3.1	9.5 ± 5.3	895.6 ± 55.2
	KMP01D	351.9 ± 11.9	201.3 ± 10.9	268.7 ± 9.0	400.7 ± 10.6	461.3 ± 11.3	10.1 ± 3.8	18.9 ± 6.9	871.4 ± 38.5
	Vit D3	369.9 ± 9.4	213.1 ± 8.6	292.5 ± 9.4	413.1 ± 11.5	499.9 ± 16.9	7.9 ± 4.2	9.1 ± 6.4	896.4 ± 58.2
	KMP01D + Vit D3	351.8 ± 10.4	205.4 ± 9.3	205.8 ± 10.1	402.1 ± 10.9	432.1 ± 14.2	18.6 ± 4.9	18.1 ± 6.1	873.5 ± 66.1

*IFN, interferon; IGF, insulin-like growth factor; IL, interleukin; PBMCs, peripheral blood mononuclear cells; TNF, tumor necrosis factor; UICC, union for international cancer control*.

**Figure 4:**
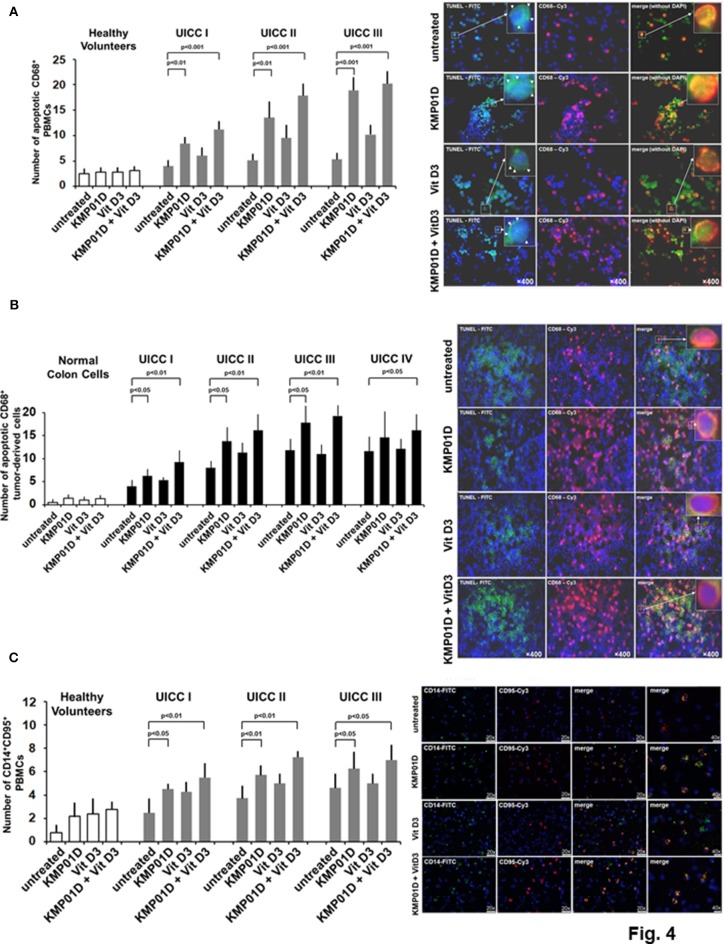
Analysis for apoptosis of peripheral blood mononuclear cells (PBMCs) and tumor-derived cells from healthy volunteers (*n* = 12) and colorectal cancer (CRC) patients (*n* = 12 for each stage). Increased apoptosis of **(A)** CD68^+^ PBMCs [representative TUNEL assay immunofluorescence staining of Union for International Cancer Control (UICC) stage III], **(B)** CD68^+^ tumor-derived cells [representative terminal deoxynucleotidyl transferase dUTP nick end labeling (TUNEL) assay immunofluorescence staining of UICC stage III], and **(C)** CD14^+^CD95^+^ PBMCs [representative immunofluorescence staining of UICC stage III for expression of CD95 (i.e., Fas)] following incubation with KMP01D. An enhanced effect was observed following incubation with the combination of KMP01D and vitamin D3. All images were obtained at 200 × or 400 × magnification. TUNEL-fluorescein isothiocyanate (FITC), green; anti-CD68-cyanine 3 (Cy3), pink; CD14-FITC; CD95-Cy3.

Apoptosis is a highly regulated process that is critical for maintaining homeostasis in multicellular organisms. In this study, we assessed the effect of KMP01D and vitamin D3 on apoptosis in *ex vivo* PBMCs and tumor-derived cells using the TUNEL assay. Our results demonstrated that incubation of PBMCs with KMP01D alone or in combination with vitamin D3 resulted in a more than 30% increase in apoptotic CD68^+^ cells (Figure 4A; representative example from UICC stage III patient; KMP01D vs. untreated cells: UICC I–II: *p* < 0.01, UICC III: *p* < 0.001; combination of KMP01D and vitamin D3 vs. untreated cells: UICC I–III: *p* < 0.001). These data suggest that the combination of KMP01D and vitamin D3 has anti-inflammatory effects attributable to the induction of apoptosis in immune cells. To gather insight into the type of apoptosis pathway involved, we analyzed the expression of the surface cell death receptor Fas, a marker of the extrinsic pathway of apoptosis also known as CD95 (33), to determine if it is involved in the apoptosis of CD14^+^ PBMCs. The cells were analyzed for the expression of CD14 and CD95 by immunofluorescence. Elevated CD14^+^CD95^+^ cell expression was observed in PBMCs of patients following *ex vivo* incubation with KMP01D and the combination of KMP01D and vitamin D3 (Figure 4C; representative example of UICC stage III; KMP01D vs. untreated cells: UICC I–III: *p* < 0.05, UICC II: *p* < 0.01; combination of KMP01D and vitamin D3 vs. untreated cells: UICC I–II: *p* < 0.01, UICC III: *p* < 0.05).

**Figure 5:**
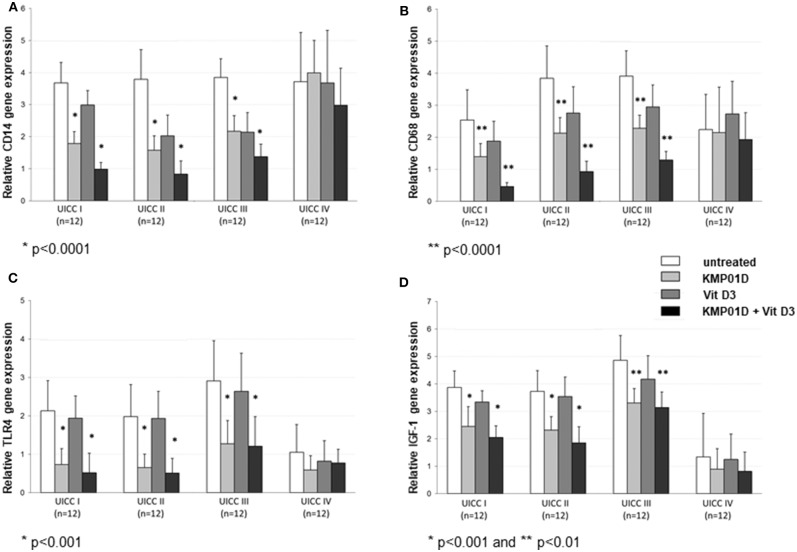
RT-qPCR analysis of cell fractions from tumor tissues showing decreased **(A)** CD14, **(B)** CD68, **(C)** Toll-like receptor (TLR)4, and **(D)** insulin-like growth factor (IGF)-1 gene expression in tumor-derived cells of Union for International Cancer Control (UICC) stage I–IV patients (*n* = 12 for each stage) following incubation with KMP01D and the combination of KMP01D and vitamin D3 compared with untreated cells. (CD14: UICC I–III: ^*^p < 0.0001 and ^*^p < 0.0001; CD68: UICC I–III: ^**^p < 0.0001 and ^**^p < 0.0001; TLR4: UICC I–III: ^*^p < 0.001 and ^*^p < 0.001; IGF-1: UICC I–II: ^*^p < 0.001 and ^*^p < 0.001, UICC III: ^**^p < 0.01 and ^**^p < 0.01, respectively). An enhanced effect on decreasing CD14 and CD68 expression was observed after incubation with the combination of KMP01D and vitamin D3 (UICC I–III).

KMP01D, both alone and in combination with vitamin D3, additionally resulted in enhanced apoptosis of tumor-derived CD68^+^ cells analyzed by the TUNEL assay (Figure 4B; representative example of UICC stage III patient; KMP01D vs. untreated cells: UICC I–III: *p* < 0.05; combination of KMP01D and vitamin D3 vs. untreated cells: UICC I–III: *p* < 0.01, UICC IV: *p* < 0.05). Moreover, gene expression of CD68 was decreased in all investigated cell fractions from tumor tissues (UICC stages I–III) when treated with KMP01D alone or in combination with vitamin D3 (KMP01D vs. untreated cells: UICC I–II: *p* < 0.0001; combination of KMP01D and vitamin D3 vs. untreated cells: UICC I–III: *p* < 0.0001). Gene expression of CD14 was likewise decreased in all investigated cell fractions from tumor tissues (UICC stages I–III) when treated with KMP01D alone or in combination with vitamin D3 (KMP01D and combination of KMP01D and vitamin D3 vs. untreated cells: UICC I–III: *p* < 0.0001). An enhanced effect of KMP01D and vitamin D3 was observed by decreasing the expression of both CD14 and CD68 (UICC stages I–III) (Figures 5A,B).

We next analyzed gene expression of TLR4 and IGF-1, both known to be expressed by tumor-infiltrating mononuclear cells and to a certain extent by tumor cells. TLR4 expression was decreased in tumor-derived cells incubated with KMP01D alone and in combination with vitamin D3 (KMP01D and combination of KMP01D and vitamin D3 vs. untreated cells: UICC I–III: *p* < 0.001; Figure 5C). Incubation of tumor-derived cells with KMP01D alone and in combination with vitamin D3 also resulted in downregulated IGF-1 expression (KMP01D and combination of KMP01D and vitamin D3 vs. untreated cells: UICC I–II: *p* < 0.001, UICC III: *p* < 0.01; Figure 5D).

## Discussion

Increasing evidence suggests a pivotal role of intestinal microbiota in the development and progression of CRC (33–37). Studies have shown that the relationship between intestinal microbiota and CRC is related to changes in composition and underlying microbial functions as shown in metagenome-wide association studies (36, 38–43). Dysbiosis of intestinal microbiota increases the release of endotoxins by gram-negative bacteria (e.g., *E. coli*) frequently amplified in CRC patients and together with a compromised gut barrier results in the leakage of endotoxins into the circulation. This process leads to the generation of low-grade and repeated inflammatory processes which negatively influence CRC patient prognosis as evidenced by previous outcome studies (44–47). Endotoxin release in a compromised gut triggers an inflammatory cascade following binding to TLR4 and CD14 which results in the upregulation of TNFα expression (14). Several studies, including those using Lactobin, have yielded results supporting the use of colostrum preparations in the treatment of infections resulting from dysregulated bacterial toxin release (17, 19–24, 48). Compared to other products, Lactobin has a notable (pre-)clinical background. The product has been shown to ameliorate hemorrhagic diarrhea in infants infected with Shiga toxin-producing *E. coli* (24). Preclinical studies and a subsequent clinical phase II placebo-controlled trial in which Lactobin was administered to patients undergoing coronary bypass surgery provided evidence in line with our results. The orally given colostral immunoglobulins and other immune-active ingredients in Lactobin (i.e., lactoferrin, vitamins, and other proteins) reduced the crossing of bacterial toxins from the intra-intestinal surface into central body compartments by intraenteral endotoxin neutralization (20, 22). Another clinical study demonstrated reduced serum endotoxin levels in patients after abdominal surgery when treated orally with Lactobin (21). Moreover, mechanistic studies have established that IL-1β, IL-6, and TNFα are involved in colorectal cancer progression and that expression of these cytokines is regulated by vitamin D (49). Our results suggest additive potentiation of KMP01D on reducing inflammatory cytokine expression profiles when combined with vitamin D3. This underlines the potential supportive role of colostral components and vitamin D3 in additive anti-inflammatory immune cancer therapy (49, 50). This may be of particular significance in UICC stage I–III CRC patients with underlying inflammatory responses as reflected by our results. UICC stage IV individuals with high tumor burden frequently demonstrate immune exhaustion which can result in comparably inferior clinical outcomes and may therefore explain our non-significant results in highly advanced tumor patients in contrast to stage I–III patients with early and localized cancers. Several groups over the last two decades have also observed elevated inflammatory cytokine and chemokine profiles in the serum of patients with solid cancers. Significantly elevated expression of IL1-β, IL-6, IL-8, IL-10, IL-17, TNF-α, TGF-β, IFN-γ, and C-X-C motif chemokine ligand 1 (CXCL1) has been seen in colorectal cancer and other solid cancers like lung cancer (51–55).

Our study shows that the expression of TLR4 was reduced in PBMCs and tumor-derived cells from CRC patients following *ex vivo* incubation with KMP01D. Furthermore, KMP01D alone and in combination with vitamin D3 effectively reduced the expression of inflammatory cytokines in CRC patient-derived PBMCs. Conversely, anti-inflammatory cytokine expression was increased in the PBMCs of UICC stage I–III patients after incubation with KMP01D alone and in combination with vitamin D3. Expression of IGF-1 has also been found in tumor-associated macrophages in CRC and other solid cancers, and its function associated with tumor cell proliferation, migration, and angiogenesis (56, 57). Moreover, previous studies have shown for IGF-1 to function in the reduction of apoptosis (53, 58) and for the corresponding receptor (IGF-1R) to demonstrate stage-dependently increased expression in metastatic colorectal tumors and to be significantly correlated with poor overall survival (54, 59). Our data confirm that IGF-1 levels are stage-dependently increased in PBMCs from CRC patients and that KMP01D decreases IGF-1 expression. Such results are in line with recent studies and suggest that by downregulating IGF-1, KMP01D could support the reversal of IGF-1-mediated anti-apoptotic effects on immune cells. The anti-inflammatory potential of KMP01D was likewise observed with CD14^+^CD68^+^ cells. We found relative expression of CD14 and CD68 to be downregulated in PBMCs and tumor-derived cells following incubation with KMP01D. Flow cytometric analysis revealed that incubation of PBMCs with the combination of KMP01D and vitamin D3 decreased the percentage of CD14^+^CD68^+^ cells. Increasing evidence supports the notion that a systemic inflammatory response is a pivotal determining factor of outcome in cancer patients reflected by low LMR which has been correlated with shortened overall survival in several malignancies including CRC (4, 5). The observed action of KMP01D and vitamin D3 could mitigate inflammatory cytokine expression and may therefore positively influence the LMR in patients with progressed CRC and an impaired antitumor immune response, particularly early after surgical removal of the primary tumor and following systemic chemotherapy. Accordingly, KMP01D and vitamin D3 together may indirectly serve to increase T effector cell responses to target tumor cells in occult distant metastases.

The binding of endotoxins to TLR4 and CD14 activates an inflammatory response and contributes to the dysregulation of mononuclear cell apoptosis (13, 14). Furthermore, *E. coli*, one strain of gram-negative bacteria implicated in CRC and detected within the epithelial surface, can persist in macrophages following endocytosis and has been shown to prevent phagocytosis-induced death of these cells via classical anti-apoptotic NF-κB signaling (15). As such, we analyzed the apoptotic signaling of PBMCs from CRC patients. Our results indicate that KMP01D promotes apoptosis of CD68^+^ cells in PBMCs and tumor-derived cells as demonstrated by our TUNEL assay. To gain insight into the apoptotic pathway involved in these cells, we looked for CD95 (i.e., Fas) expression, a marker characteristic of the extrinsic pathway of apoptosis (60). PBMCs of CRC patients incubated with KMP01D showed elevated CD95 expression, suggesting that apoptosis of these cells proceeds via the extrinsic pathway of apoptosis and thereby contributes to downregulation of inflammatory cytokine expression.

Intravenous immunoglobulins (IVIGs) have been used for more than six decades, notably as an adjunct therapy in patients with sepsis and recurrent inflammatory conditions of varying microbiological etiologies. Due to the wide spectrum of diseases and immune disorders with an indication for IVIGs, their consumption for licensed and unlicensed/off-label uses is multiplying every year in North America, Europe, and Asia. Owing to their stimulatory effects on certain subpopulations of T cells (e.g., regulatory T cells), as shown in recent studies, IVIGs are currently envisioned as a potential strategy in situations of severe lymphopenia, seen in cancer patients after chemo- and radiotherapy (61). Not unlike PVIGs, insights from sepsis studies have shown that the IVIGs mediate their immunomodulatory and anti-inflammatory effects through Fcγ receptors, enabling immune cells to phagocytose opsonized pathogens via Fcγ receptor pathways (62). IVIGs can neutralize foreign antigens that have entered the circulation via epithelial surfaces (e.g., mostly gut and lung). IVIGs are blood-derived products from a pool of more than 1,000 donors (frequently more than 10,000 donors), providing a broad spectrum of opsonic and neutralizing IgG antibodies against a variety of microbial antigens and epitopes. Consequently, the IgG antibody content varies with each product batch, primarily due to differences in the local pathogen ecology of donor exposure. Among several products on the market only one preparation, Pentaglobin^®^ (Biotest, Germany), is IgM-enriched and particularly effective in ameliorating gram-negative inflammatory responses. In addition, the manufacturing process necessitates several (non-)chemical processes for virus (hepatitis and HIV) reduction which contributes to high costs. The value of IVIG therapy remains under discussion particularly when considering costs, variations in immunoglobulin content (IgG and IgM), the type of predominant inflammation in a patient (gram-negative or gram-positive), and differentially reported clinical efficacy. In contrast to IVIGs, KMP01D is produced from a well-defined pool of bovine-derived colostra with a multitude of PVIGs. The colostrum preparation is available in high quantities at comparably low costs and contains immunoglobulins against predominantly gram-negative bacterial-derived O-chain antigens. With a significant endotoxin neutralization capacity, Lactobin acts at the epithelial surface of the gut as evidenced in several clinical trials (17, 19–22, 48). Lactobin mitigates endotoxin release and leakage, particularly in a gut with an inflammation-mediated compromised intestinal epithelial barrier.

In the present study, we sought to analyze, for the first time, the *ex vivo* effects KMP01D on PBMCs and tumor-derived cells of UICC stage I–IV CRC patients. The experiments here set forth showed that KMP01D significantly and stage-dependently decreased inflammatory cytokine expression in PBMCs and promoted apoptosis of immune cells from our cohort of CRC patients. Collectively, our findings are in line with previous preclinical and clinical trial data demonstrating the endotoxin neutralizing capacity of the colostrum preparation. KMP01D may well serve as an adjunct treatment strategy in the gut to reduce upregulated inflammatory cytokine responses and to diminish mononuclear cell activation. Our results emphasize the need for clinical studies in patients with CRC having disturbed inflammatory immune responses.

## Data Availability Statement

The datasets generated for this study are available on request to the corresponding author.

## Ethics Statement

The studies involving human participants were reviewed and approved by Regional Ethics Committee of the University Wuerzburg. The patients/participants provided their written informed consent to participate in this study.

## Author Contributions

MG, AW-G, and RL designed the study. MG and AW-G analyzed the data. MG, AW-G, KN, RL, and L-LH interpreted the results. MG, AW-G, and KN drafted the manuscript. L-LH, AW-G, and MG edited the manuscript. All authors have approved the final version of the manuscript and agree to be accountable for all aspects of the work.

## Conflict of Interest

MG, RL, and AW-G have a patent on LactobinN/Vitamin D3 (PCT/EP2013/075287) and a pending patent in USA (Appl. No. 15/100,730). The remaining authors declare that the research was conducted in the absence of any commercial or financial relationships that could be construed as a potential conflict of interest.

## Supplementary Material

Supplementary Figure 1 Representative flow cytometric analysis of monocyte frequency in PBMCs from a healthy volunteer (control) and UICC stage I–IV CRC patients. Monocyte frequency is shown for **(A)** untreated PBMCs, **(B)** PBMCs incubated with KMP01D, **(C)** PBMCs incubated with vitamin D3, and **(D)** PBMCs incubated with a combination of KMP01D and vitamin D3. Decreased monocyte (CD14^+^CD45^+^) frequency was observed following incubation of PBMCs of UICC stage I-III CRC patients with KMP01D. The combination of KMP01D and vitamin D3 demonstrated enhanced effects. (Mon., Monocytes, orange; Gran., Granulocytes, pink; Lymph., Lymphocytes, blue).
