# Supplementary figures and images for: KMP01D Demonstrates Beneficial Anti-inflammatory Effects on Immune Cells: An ex vivo Preclinical Study of Patients With Colorectal Cancer
Source: Front Immunol. 2020 Apr 30;11:684. doi: 10.3389/fimmu.2020.00684 (PMC7205007; doi:10.3389/fimmu.2020.00684)

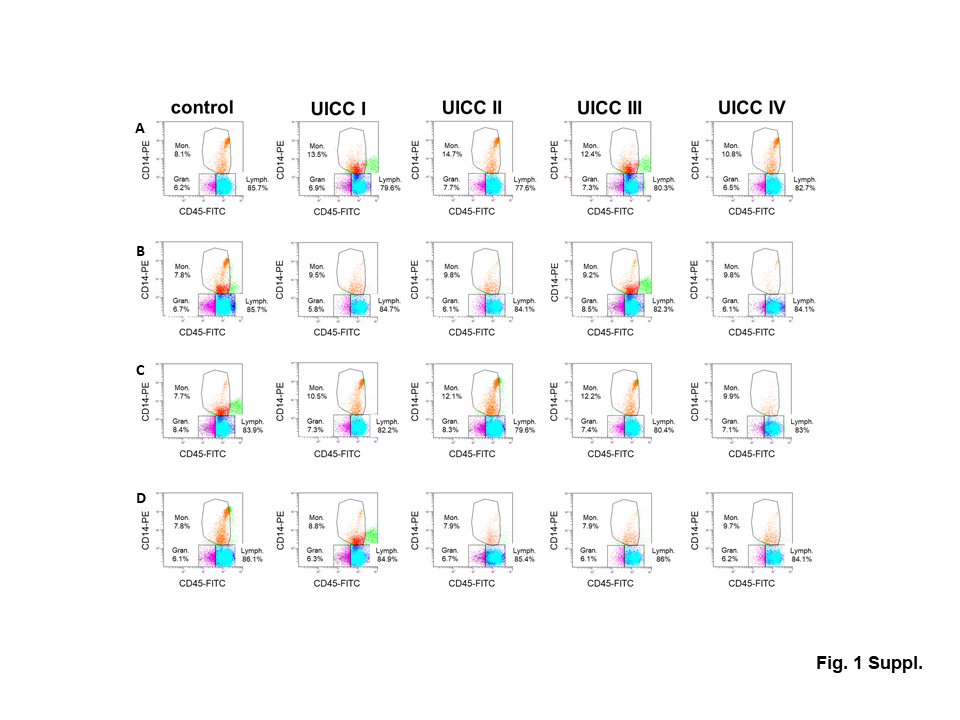

Supplement: Supplementary Figure 1 — Representative flow cytometric analysis of monocyte frequency in PBMCs from a healthy volunteer (control) and UICC stage I–IV CRC patients. Monocyte frequency is shown for (A) untreated PBMCs, (B) PBMCs incubated with KMP01D, (C) PBMCs incubated with vitamin D3, and (D) PBMCs incubated with a combination of KMP01D and vitamin D3. Decreased monocyte (CD14+CD45+) frequency was observed following incubation of PBMCs of UICC stage I-III CRC patients with KMP01D. The combination of KMP01D and vitamin D3 demonstrated enhanced effects. (Mon., Monocytes, orange; Gran., Granulocytes, pink; Lymph., Lymphocytes, blue). [file Image_1.TIF]
